# A Case of Cancer, Successfully Treated by Arsenic

**Published:** 1802-05

**Authors:** Geo. Custance

**Affiliations:** Kidderminster


### *A Case of Cancer, successfully treated by Arsenic.*

To the EDITORS of the MEDICAL and PHYSICAL JOURNAL;

Gentlemen,

THE following Case has nothing particular in it, only as affording an additional evidence to that already laid before the public by other professional men, of the power which Arsenic sometimes possesses in curing an open Cancer. I shall content myself myſelf with ſimply ſtating the Caſe, and making two remarks, in order that, if you think them of ſufficient importance to inſert in your uſeful Miſcellany, they may not occupy too much room in it.

Mary Millaird, between forty and fifty years of age, the wife of a carpet-weaver in this town, was admitted to the Kidderminſter Diſpenſary on the 13th of January laſt. She had been near two years afflicted with a cancerous tumour, ſituated near the middle of the fore-arm, on the outſide. She informed me, that from her birth ſhe had had a ſmall mole-ſpot in that place, which ſhe one day unfortunately knocked againſt a door and made it bleed, from which time it grew ſore; and when I firſt ſaw it, it was as big and as round as a large muſhroom. Its ſurface was very irregular; it bled upon the ſlighteſt touch, was extremely painful, and the ſmell of it intolerably offenſive. From the abſorption of matter, there was an enlarged gland, about two inches above the ulcer, three in the axilla, and ſeveral along the ſide of the neck. The advice of different practitioners had afforded no relief. I immediately ſprinkled the ulcer over with finely powdered arſenic. In about a week the whole tumour ſloughed off, and left a clean ſurface at the bottom. I then dreſſed the arm daily with ung. baſilic. nigr. It granulated very kindly, and is now perfectly healed. The poor woman expreſſes great happineſs in being freed from ſo loathſome a ſore.

I remark, in the firſt place, that this Caſe was clearly, in its origin, entirely local, and that in conſequence of the blow the part put on the ſpecific action, which induced a cancer. Secondly, it is to be lamented that the arſenic was not applied before the glandular ſwellings took place, as, in that caſe, a complete cure would have been obtained; but now further miſchief may hereafter enſue, although the primary local diſeaſe is quite removed.

I am, &c.

*Kidderminster, April* 15, 1802. GEO. CUSTANCE.

P. S. Permit me to embrace this opportunity of thanking Mr. Reece for his obliging compliance with my requeſt of a Drawing of the Tooth Inſtrument he recommended. Your Readers will probably recollect that my doubts were reſpecting its power of raiſing a Tooth *perpendicularly*, and not as to any other *improvement* it might be upon former Inſtruments.

